# Inhibition of placental growth factor improves surgical outcome of glaucoma surgery

**DOI:** 10.1111/jcmm.12151

**Published:** 2013-10-09

**Authors:** Tine Bergen, Bart Jonckx, Karolien Hollanders, Davine Sijnave, Sarah Velde, Evelien Vandewalle, Lieve Moons, Jean-Marie Stassen, Ingeborg Stalmans

**Affiliations:** a Laboratory of Ophthalmology, KU LeuvenLeuven, Belgium; b Research and Development, ThromboGenics NVHeverlee, Belgium; c Department of Ophthalmology, University Hospitals LeuvenLeuven, Belgium; d Department of Biology, KU LeuvenLeuven, Belgium

**Keywords:** PlGF, glaucoma surgery, wound healing, inflammation, angiogenesis, fibrosis

## Abstract

Excessive post-operative wound healing with subsequent scarring frequently leads to surgical failure of glaucoma filtration surgery (trabeculectomy). We investigated the hypothesis that placental growth factor (PlGF) plays a role in post-operative scar formation, and that it therefore may be a target for improvement of filtration surgery outcome. ELISA experiments showed that PlGF levels were significantly increased in aqueous humour of glaucoma patients and after VEGF treatment, which may indicate an important contribution of this growth factor to wound healing after trabeculectomy. Using a mouse model of glaucoma filtration surgery, we were able to show that intracameral injection of a previously characterized anti-PlGF antibody (ThromboGenics NV) significantly improved surgical outcome by increasing bleb survival and bleb area. This was associated with a significant reduction in post-operative proliferation, inflammation and angiogenesis during the first post-operative days after surgery, and with a decrease in collagen deposition at later stages. Furthermore, inhibition of PlGF seemed to be more effective than anti-VEGF-R2 treatment in improving surgical outcome, possibly *via* its additional effect on inflammation. These results render PlGF an appealing target for ocular wound healing and point to potential therapeutic benefits of PlGF inhibition for the prevention of surgical failure.

## Introduction

Trabeculectomy is a widespread, well-studied surgical method of intraocular pressure (IOP) reduction in the management of glaucoma 1–2. Subconjunctival fibrosis and wound healing form the major reasons for insufficient aqueous filtration and surgical failure 3. The process of wound healing is a cascade of different processes that are closely linked, including coagulative and inflammatory phases, followed by proliferation and repair phases and finally, the remodelling phase 4. Various growth factors are known to be involved in post-operative wound healing, *e.g*. transforming growth factor (TGF)-β, fibroblast growth factor (FGF) and VEGF 5,6. Importantly, a recent study of Rodriguez-Agirretxe *et al*. showed that up-regulation of TGF-β and VEGF in conjunctival biopsies of glaucoma patients was highly correlated with surgical failure, whereas up-regulation of other factors [*e.g*. interleukin (IL)-6, IL-8, matrix metalloproteinase (MMP)-1, MMP-2, *etc*.] were associated with surgical success 8. VEGF plays an important role in both physiological 9 and pathological angiogenesis 10–11. VEGF-R2 mediates most biologically relevant VEGF responses, including vascular permeability, cell migration and proliferation 12. Alternative splicing of a single VEGF gene can result in multiple isoforms, such as VEGF_121_, VEGF_165_ and VEGF_189_ 13. We have previously shown that pharmacological enhancement of trabeculectomy using VEGF inhibitors was able to significantly improve rates of surgical success. A single injection of bevacizumab (non-selective VEGF inhibitor) at the time of trabeculectomy could improve the surgical outcome by reducing post-operative angiogenesis during the initial phase, and collagen deposition at later stages in a rabbit trabeculectomy model 14. Injection(s) of pegaptanib (a selective VEGF_165_ inhibitor) improved surgical outcome less efficiently by reducing angiogenesis only, because of a retained action of VEGF_121_ and VEGF_189_, which have a more pronounced effect on fibrosis 15. Importantly, neither selective nor non-selective VEGF inhibition could reduce inflammation, an important process in post-operative wound healing. Importantly, in different animal models of wound healing, it has also been described that administration of bevacizumab does not reduce inflammation 16–19. This can presumably be partially explained by an up-regulation of placental growth factor (PlGF), a pro-inflammatory growth factor 20,21. PlGF, a VEGF-homologue, which solely binds to VEGF-R1 23, only acts on pathological angiogenesis 24 and inflammation 25 and is not involved in physiological angiogenic processes. A monoclonal anti-PlGF antibody against mouse PlGF (5D11D4), developed by ThromboGenics NV (Heverlee, Belgium), was previously shown to inhibit tumour growth in different mouse tumour models 26,27. Moreover, Van de Veire *et al*. showed a dose-dependent reduction in murine choroidal neovascularization (CNV) formation, by reducing angiogenesis and inflammation. They also demonstrated that intraocular use of the antibody was safe 28. Other studies showed a beneficial effect of the PlGF antibody in the development of atherosclerotic plaque formation 29 and liver cirrhosis 30, by its inhibitory effects on inflammation and fibrosis.

In this study, the potential therapeutic effect of a monoclonal PlGF antibody and its mechanism of action in the inhibition of wound healing after glaucoma filtration surgery were evaluated and compared with the effects of an antibody to VEGF-R2. Our findings suggest that inhibition of PlGF might be more effective in improving surgical outcome as compared with VEGF-R2 inhibition, through its additional effect on inflammation.

## Materials and methods

All procedures conformed to the tenets of the Declaration of Helsinki and written informed consent was obtained from patients after gaining approval from the institutional human ethics committee (Institutional Review Board of the University Hospitals Leuven). All animals were used in accordance with the standards in the Association for Research in Vision and Ophthalmology Statement for the Use of Animals in Ophthalmic and Vision Research. The Institutional Animal Care and Research Advisory Committee of KU Leuven approved all experimental animal procedures.

### Patients and biochemical measurements

Samples of human aqueous humour (AH) and plasma were collected from patients (*n* = 10) who underwent trabeculectomy for primary open angle glaucoma (POAG) or phacoemulsification for senile cataract without glaucoma (the control group). Glaucoma was defined as having characteristic optic disc damage (based on cup/disc ratio, thinning of neuroretinal rim, notching, disc haemorrhages, *etc*.) and visual field defects. For the diagnosis of POAG, an untreated IOP of >21 mmHg was required 31. The surgeon collected samples of AH (100–200 μl) immediately after limbal paracentesis to avoid the influence of intraocular trauma/surgery. Blood was collected in ethylene diamine tetracetic acid (EDTA)-coated tubes and centrifuged for 15 min. at 1248 × *g* AH and plasma samples were stored immediately at −80°C until analysis. PlGF protein levels were analysed in AH and plasma samples by using a double-antibody ‘sandwich’ ELISA (R&D Systems, Minneapolis, MN, USA), with a detection limit of 15.6 pg/ml. Concentrations were expressed as pg/ml.

### Cell culture and proliferation assay

Murine Tenon’s tissues were obtained from C57BL/6J mice before filtration surgery by dissecting a piece of the Tenon’s capsule. Murine Tenon fibroblasts (MTF) were prepared by dissociating these freshly dissected tissues mechanically and enzymatically. Tissue pieces were cut, trypsinized for 30 min. and centrifuged at 312 × *g* for 5 min. Primary Tenon fibroblasts were propagated in DMEM medium (Invitrogen Corporation, Carlsbad, CA, USA), supplemented with 10% foetal bovine serum (FBS; Thermo Fisher Scientific, Rochester, NY, USA), 2 nM L-glutamate, 100 U/ml penicillin, 100 μg/ml streptomycin (all from Invitrogen). Subconfluent MTF were trypsinized and were seeded in 96-well plates at an initial density of 5 × 10^3^ cells/well in 100 μl complete medium. In one series of experiments, the cells were serum starved (medium supplemented with 0.1% FBS) overnight, 6 hrs after cell seeding. The medium of MTF was changed to fresh serum-free medium containing recombinant murine PlGF and VEGF-A (further referred to as VEGF; 10, 25, 50 and 100 ng/ml; both from R&D Systems). In another series of experiments, the complete medium of MTF was replaced by complete medium, pre-incubated with PlGF and VEGF (50 ng/ml; R&D Systems) in presence of anti-PlGF antibody (5D11D4), anti-VEGF-R2 antibody (DC101) or an irrelevant mouse antibody (1C8) (0.1, 1, 10 and 100 μg/ml). Forty-eight hours after growth factor or antibody administration, cell proliferation was assessed in all experiments by using the WST-1 Cell Proliferation Assay System (Roche Diagnostics, Mannheim, Germany). Complete or serum-free medium was used as controls.

### Quantitative real time RT-PCR

RNA from MTF was isolated by using the RNeasy Minikit (Qiagen, Valencia, CA, USA) and quantitative RT-PCR was performed, as described previously 24. Expression was normalized to that of the housekeeping gene β-actin. Following forward (for) and reverse (rev) primers and probes (pro) labelled with a fluorescent dye (FAM) and quencher (TAMRA) were used. Murine β-actin: for 5′-AGA-GGG-AAA-TCG-TGC-GTG-AC-3′; rev 5′-CAA-TAG-TGA-TGA-CCT-GGC-CGT-3′; pro 5′-CAC-TGC-CGC-ATC-CTC-TTC-CTC-CC-3′. Murine PlGF: for 5′-CCC-TGT-CTG-CTG-GGA-ACA-AC-3′; rev 5′-CAG-TAG-CTG-CGA-CCC-CAC-A-3′; pro 5′-ACA-GAA-GTG-GAA-GTG-GTG-CCT-TTC-AAC-3′. Murine VEGF: for 5′-TGC-ACC-CAC-GAC-AGA-AGG-A-3′; rev 5′-GGC-AGT-AGC-TTC-GCT-GGT-AGA-C-3′; pro 5′-CAG-AAG-TCC-CAT-GAA-GTG-ATC-AAG-TTC-ATG-GA-3′. Murine VEGF-R1: for 5′-AGC-CCC-TCA-CCA-TGG-AAG-A-3′; rev 5′-CCG-ATG-AAT-GCA-CTT-TCT-GGA-3′; pro 5′-TTT-CCT-ACA-GTT-TCC-AAG-TGG-CCA-GAG-GC-3′. Murine VEGF-R2: for 5′-CCT-CTA-CAC-CTG-CCA-GGC-C-3′; rev 5′-TTC-CTG-GGC-ACC-TTC-T AT-TAT-GAA-3′; pro 5′-TTG-GCT-GTG-CAA-GAG-CGG-AGA-CG-3′.

### Rabbit model for glaucoma surgery

New Zealand rabbits (*n* = 5; 12–14 weeks old) were obtained from the animal facility of KU Leuven. General anaesthesia was induced by intramuscular injection of 50 mg/ml ketamin (Ketalar, Pfizer, Ann Arbor, MI, USA) and 2% sedative (Rompun, Bayer Health Care, Pittsburgh, PA, USA). Filtration surgery was performed on both eyes by using a technique as previously described 14. Immediately after surgery, one eye was injected with 25 mg/ml bevacizumab and the other eye was used as a control and received an injection of 0.9% NaCl. For each eye, 200 μl was injected into the anterior chamber and 100 μl was injected subconjunctivally into the filtration bleb, based on our previous study 14. Samples of AH and blood were obtained from the rabbits the day before and on days 3, 7, 14 and 30 after surgery. Blood was collected in EDTA-coated tubes and centrifuged for 15 min. at 1248 × *g* AH and plasma samples were stored immediately at −80°C until analysis. The levels of PlGF protein were analysed with a quantitative sandwich enzyme immunoassay technique with a detection limit of 1.0 pg/ml (E04P0018; BlueGene, Shanghai, China). Plasma of a pregnant rabbit on day 25 was used as a positive control, since we showed that it contains high PlGF levels. Concentrations were expressed as pg/ml.

### Mouse model of glaucoma filtration surgery

C57BL/6J mice (8–10 weeks old, Charles River Laboratories, Lyon, France) were anaesthetized with an intaperitoneal injection of 10 times-diluted (60 mg/kg final dose) sodium pentobarbital (Nembutal, 60 mg/ml; CEVA Sante Animale, Brussels, Belgium). Before surgery, IOP was measured in both eyes with a tonometer (TonoLab; Technop, Espoo, Finland); 15 recordings per eye were averaged. Filtering surgery was performed on both eyes by using a technique that has been described previously and that results in a filtering bleb 32–33. Immediately after surgery, mice were divided into different groups and their eyes were injected with either 5D11D4 or DC101; an isotype-matched control antibody (1C8) was used as a negative control. The injections were performed by using an analytic science syringe (SGE Analytic Science) and glass capillaries with a diameter of 50–70 μm at the end, controlled by the UMP3I Microsyringe Injector and Micro4 Controller (all from World Precision Instruments Inc., Hertfordshire, UK).

In the first experiment (*n* = 10 eyes for all groups), mice were divided into different groups to investigate the most optimal administration route of the PlGF antibody. Immediately after surgery, the PlGF inhibitor (5.2 μg) was intracamerally (AC) injected in the first group of mice, subconjunctivally (SC) in the second group and the third group received an intravitreal (IV) injection of 5D11D4. The isotype-matched control antibody (1C8) was used in every group as a negative control. In the second experiment (*n* = 20 eyes for all groups), 5D11D4 (5.2 μg) was administered in the first group of mice and a second group received DC101 injections (6.2 μg) as positive control. The third group of mice was treated with 1C8 (4.8 μg) and was used as a negative control. For each eye, 1 μl was injected into the AC. In a third experiment (*n* = 10 eyes for all groups), repeated intracameral injections of antibodies (1 μl) were given in the same concentration on days 0, 4 and 10. These concentrations and time-points were based on previous intravitreal injections of the anti-PlGF antibody performed by Van de Veire *et al*. 28. Mice were clinically examined on day 1 after surgery and then every 2 days until they were killed. The IOP and bleb area were analysed under topical anaesthesia. Commercial software (KS300; Zeiss, Oberkochen, Germany) was used to determine the bleb size on bleb images of mice. These pictures were taken with a digital camera (Canon PowerShot S50) by using a 3× optical zoom lens at a magnification of 4×. Bleb survival was taken as the end point of the study, while bleb failure was defined as the appearance of a scarred and flat bleb at two consecutive measurements.

### Histology, immunohistochemistry and immunofluorescent stainings

Primary Tenon fibroblasts were identified based on their morphology, on their immunopositivity after immunostaining for the mesenchymal cell marker vimentin and on the absence of staining for the epithelial cell marker cytokeratin. Briefly, the cells were plated in a 12-well plate (10 × 10^4^ cells per well) on a cover slip and were grown overnight. The next day, the cells were fixed with 4% paraformaldehyde (PFA) for 30 min. at room temperature (RT), permeabilized, and blocked with PBS - 0.1% Triton X-100 - 0.3% BSA and 5% rabbit serum for 1 hr. Subsequently, the cells were incubated overnight at 4°C with the primary antibodies. Murine antibodies against vimentin (1/100; Sigma-Aldrich, V5255) and cytokeratin (1/200; SC81714) and goat antibodies against murine VEGF (1/50; SC1836) and PlGF (1/50; SC1882) were used (all from Santa Cruz Biotechnology Inc). Immunofluorescent labelling was visualized after incubation for 1 hr at RT with appropriate secondary antibodies conjugated to Alexa Fluor-568 and Alexa Fluor-488 (1/200; Invitrogen). Finally, the cover slips were mounted with Prolong Gold with 4′,6-diamidino-2-phenylindole (DAPI; Molecular Probes, Eugene, OR, USA). To confirm the specificity of the primary antibodies, staining of cells incubated without primary antibody was used as negative control. As a positive control for the cytokeratin staining, murine melanoma B16/F10 cells (provided by ThromboGenics, Heverlee, Belgium) were used 34.

On post-operative days 8 and 14 after surgery, mice were killed by cervical dislocation. Both eyes were enucleated and whole eyes were fixed in 1% PFA overnight. Serial paraffin sections were cut at 7 μm thickness in five series of five glass slides. Haematoxylin and eosin staining was performed on the first slide from each series to localize the bleb (positive area of analysis) and the rest of the eye (negative area of analysis). Proliferation was checked by performing a Ki67 staining. The slides were incubated overnight with a goat anti-murine Ki67 antibody (1/20; SC7846, SantaCruz Biotechnology Inc). The next day, immunofluorescent labelling was visualized by using a rabbit anti-goat secondary antibody, conjugated to Alexa Fluor-488 (1/200). Inflammation was analysed by a CD45 staining and a CD31 staining was performed to check the blood vessels. The mice samples were incubated overnight with rat antimouse CD45 antibody (1/100; 553076; Pharmingen, Erembodegem, Belgium) or rat antimouse CD31 (1/500; 557355; Pharmingen), respectively. The following day, the bound antibodies were visualized by using the Perkin Elmer kit (Renaissance TSA− Indirect; NEL704A; Waltham, MA, USA) and with cyanin 3 as fluorophore. Deposition of collagen was analysed in both groups by Sirius Red staining.

### Imaging and analysis

Images were obtained by using a microscope (Leica Microsystems, Wetzlar, Germany), equipped with a digital camera (Axiocam MrC5; Carl Zeiss), at a magnification of 20× and a resolution of 2584 × 1936 pixels. Morphometric analyses were performed with commercial software (KS300; Zeiss). As described above, the bleb was localized on the first slide of each series, based on the haematoxylin and eosin staining. The consecutive slides, on which the bleb was located, were used to perform the different (immuno)histological stainings. Analysis of the different processes of wound healing was only performed in the bleb (five sections/eye). The *in vivo* proliferation was analysed by counting the number of Ki67-positive cells as a percentage of the total number of cells with nuclear staining (DAPI) in the bleb 35. The density of blood vessels and leucocytes was determined by calculating, respectively, the CD31-positive and the CD45-positive area as a proportion of the bleb area. Deposition of collagen was determined by measuring the percentage of the collagen positive area in the bleb area under polarized light.

### Statistical analysis

All *in vitro* and immunomorphometric data were analysed by using the Student’s *t*-test for independent samples. Data at individual time-points were analysed by using mixed model analysis for repeated measures (with GraphPad Prism 5). Kaplan–Meier survival analysis was performed for bleb failure by using the log-rank test. *P* < 0.05 was considered to be statistically significant. Data are represented as mean ± SEM, unless otherwise stated.

## Results

### Up-regulation of PlGF in AH of glaucoma patients and after VEGF-treatment

**Figure 1:**
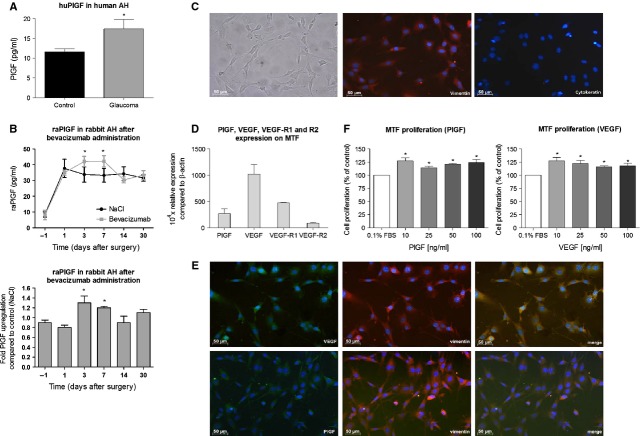
Placental growth factor (PlGF) plays an important role in scar formation *in vitro*. (A) PlGF levels were up-regulated in aqueous humour of glaucoma patients (*P = 0.03 *versus* control participants; *n* = 10 per group). (B) Bevacizumab treatment induced a significant increase in aqueous PlGF levels on day 3 (1.3-fold) and 7 (1.2-fold), as compared with control eyes injected with NaCl (*P < 0.05 *versus* control; *n* = 5). (C) The cultures of primary murine Tenon fibroblasts (MTF) clearly show an adherent homogeneous morphology of spindly, generally flat, elongated shaped cells (left panel). The cells are immunopositive for vimentin (red; middle panel), but do not show any staining for the epithelial cell marker, cytokeratin (red; right panel). Scale bar: 50 μm. (D) VEGF, PlGF and their receptors (VEGF-R1 and -R2) were expressed by MTF. The mRNA levels were normalized to that of the house keeping gene β-actin. (E) Tenon fibroblasts were immunopositive for vimentin (red; middle panels) and VEGF and PlGF (green; left panels). VEGF and PlGF both colocalized with vimentin in the cytoplasm of the cells (merge; right panels). 4′,6-diamidino-2-phenylindole (blue) was used a nuclear counter staining. Scale bar: 50 μm. (F) Addition of recombinant murine VEGF and PlGF (10–100 ng/ml) significantly augmented MTF proliferation [*P < 0.05 *versus* the control medium, containing 0.1% FBS (white bar)].

Glaucoma patients might be predisposed to more aggressive scarring after filtration surgery because of the presence of different growth factors in their AH 36. In previous work, we already showed that VEGF was significantly up-regulated in AH of glaucoma patients 14. Aqueous levels of PlGF in glaucoma patients, however, are still unknown. Therefore, PlGF concentrations in AH samples were analysed by ELISA, and were found to be significantly up-regulated by 40% in glaucoma patients as compared with the controls (*n* = 10 per group, *P* = 0.03; Fig. 1A). To elucidate whether PlGF in AH originates from the blood or is locally produced, AH and plasma concentrations were compared. No significant differences were found in plasma levels of PlGF between patients who underwent trabeculectomy (glaucoma patients) and cataract patients (control patients) (data not shown).

We also showed in previous studies that neither selective nor non-selective VEGF inhibition could reduce inflammation, presumably because of an up-regulation of PlGF 14–15. Therefore, we investigated PlGF levels in AH and plasma of operated rabbits after bevacizumab treatment. After surgery, aqueous PlGF was significantly up-regulated in the control eyes (NaCl injection) from post-operative day 1 to day 30 as compared with the PlGF levels on the day before surgery (*n* = 5; *P* < 0.05; Fig. 1B). Moreover, bevacizumab was able to significantly enhance this post-operative PlGF up-regulation (post-operative days 3 and 7), as compared with the control eyes [1.30 ± 0.14 fold (*P* = 0.03) and 1.20 ± 0.03 fold (*P* = 0.01) respectively]. PlGF levels were found to be similar in all plasma samples, taken before or after surgery (data not shown). Plasma of a pregnant rabbit on day 25 was 18.2 ± 2.44 fold up-regulated compared with normal plasma (*P* < 0.001; positive control; data not shown).

The significant increases in PlGF levels in AH, but not in the plasma, suggest that this growth factor is produced locally and can importantly contribute to wound healing after glaucoma filtration surgery. Importantly, PlGF was also up-regulated in AH of operated rabbits after VEGF inhibition.

### Expression of VEGF, PlGF and their receptors by mouse Tenon fibroblasts

Tenon fibroblasts are regarded as the key players in the initiation of wound healing and fibrotic scar formation after trabeculectomy. Cells were isolated from mouse Tenon and after 14 days in culture, they showed an adherent homogeneous morphology of spindly, generally flat, elongated shaped cells. Moreover, all cells were found to be immunopositive for vimentin and immunonegative for the epithelial cell marker, cytokeratin. Based on these stainings and their morphology, the cells were predominantly identified as MTF cultures (Fig. 1C). Murine melanoma B16/F10 cells, which served as a positive control, were clearly immunopositive for cytokeratin (data not shown). Quantitative RT-PCR experiments were performed to elucidate the mRNA expression of VEGF, PlGF and their receptors (VEGF-R1 and – R2). Although we cannot compare their absolute expression values, our data do indicate that both growth factors and their receptors are expressed by MTF (Fig. 1D). Moreover, immunostainings showed that MTF also expressed both growth factors on protein level. Double stainings revealed that both VEGF and PlGF colocalized with vimentin in the cytoplasm of the fibroblast cells (Fig. 1E).

### Stimulation of MTF proliferation by VEGF and PlGF

Growth factors in glaucomatous AH are reported to increase fibroblast proliferation by 60% in comparison with AH of controls 36, which may lead to an increased risk of filtration failure. To determine the effect of PlGF and VEGF on proliferation of MTF, cells were grown in serum-free medium to which the growth factors were added. Murine PlGF, administered at 10, 25, 50 and 100 ng/ml, significantly increased MTF by 27%, 14%, 21% and 24% respectively (*P* < 0.05). Administration of murine VEGF induced a significant increase in MTF proliferation as compared with serum-free medium, with 27%, 22%, 16% and 18% increase for 10, 25, 50 and 100 ng/ml respectively (*P* < 0.05; Fig. 1F).

Overall, these data show that PlGF and VEGF, which are expressed in AH and by MTF, stimulate MTF proliferation and thus might play an important role in scar formation after glaucoma filtration surgery.

### PlGF and VEGF inhibition reduces MTF proliferation *in vitro*

**Figure 2:**
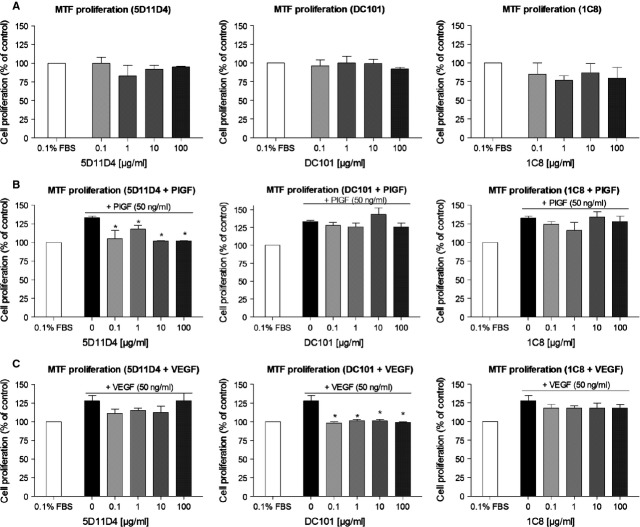
PlGF and VEGF-R2 inhibition reduces cell growth of MTF *in vitro*. (A) Administration of 5D11D4, DC101 or 1C8 did not induce any specific inhibitory effects on basal cell proliferation of MTF (*P* = NS). (B) 5D11D4 significantly inhibited PlGF-induced proliferation [*P < 0.05 *versus* 0.1% FBS + PlGF (black bar)], whereas DC101 and 1C8 did not affect MTF proliferation after PlGF stimulation (*P* = NS). (C) VEGF-induced proliferation of MTF was significantly reduced after DC101 administration [*P < 0.05 *versus* 0.1% FBS + VEGF (black bar)], while 5D11D4 and 1C8 did not have any effect (*P* = NS).

Administration of the anti-PlGF antibody 5D11D4 to MTF did not result in any inhibitory effects on their cell proliferation, which is not surprising as PlGF has not been reported to play a major role in physiological processes (*P* = NS; Fig. 2A). However, when increasing concentrations of the 5D11D4 antibody (0.1, 1, 10 and 100 μg/ml) were administered together with PlGF (50 ng/ml), PlGF-induced proliferation of MTF was reduced with 29%, 15%, 31%, 31%, respectively, as compared with control (0.1% FBS with PlGF; *P* < 0.05; Fig. 2B). To investigate the effect of VEGF inhibition on MTF proliferation, we used the well-described rat antimouse VEGF-R2 antibody DC101 37–38, as a mouse VEGF-specific monoclonal antibody is not available. We showed that unstimulated MTF proliferation was not affected after DC101 administration (*P* = NS; Fig. 2A), whereas VEGF-induced proliferation of MTF was significantly inhibited by DC101 (0.1, 1, 10 and 100 μg/ml) by 31%, 27%, 27% and 29%, respectively, as compared with control (0.1% FBS with VEGF; *P* < 0.05; Fig. 2C). An irrelevant control antibody 1C8 (0.1, 1, 10 and 100 μg/ml) did not influence (PlGF and VEGF-induced) MTF proliferation (*P* = NS for all concentrations; Fig. 2A–C). Of note, 5D11D4 did not significantly influence VEGF-induced cell proliferation, whereas DC101 administration did not affect PlGF-induced cell growth (*P* = NS; Fig. 2B and C).

Thus, we demonstrated that administration of neutralizing antibodies to murine PlGF and murine VEGF-R2 reduced, respectively, PlGF- and VEGF-induced proliferation of MTF, although dose–response effects were not found.

### Optimal route of administration of the anti-PlGF antibody

**Figure 3:**
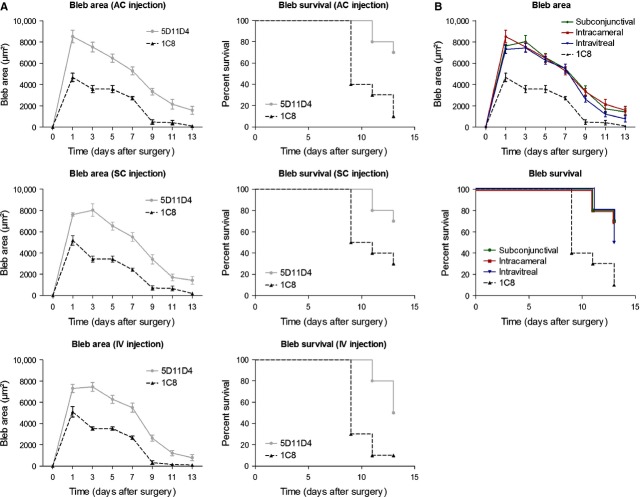
The most optimal route of administration of the anti-PlGF antibody (A) Bleb area (*P* < 0.001; *n* = 10) and bleb survival (*P* < 0.05; *n* = 10) were significantly improved after a single intracameral (AC), subconjunctival (SC) and intravitreal (IV) injection of the PlGF inhibitor, compared with their respective controls. (B) A direct comparison between the three groups showed no significant difference in bleb area and survival (*P* = NS; *n* = 10).

Previous study showed intra-ocular safety of anti-PlGF injections in the eye 28; however, the most optimal route of administration of the PlGF antibody is still uncertain. Therefore, surgical outcome after a single intracameral (AC), subconjunctival (SC) and intravitreal (IV) injection of the PlGF antibody (5D11D4, 5.2 μg) was compared. Bleb area and bleb survival were analysed until 14 days after surgery and showed that the three administration routes of the PlGF antibody were able to significantly improve bleb area (*n* = 10; *P* < 0.001) and bleb survival (*n* = 10; *P* < 0.05) compared with their respective controls (1C8; 4.8 μg; Fig. 3A). A direct comparison among the three groups showed no significant difference in bleb area and survival (*n* = 10; *P* = NS; Fig. 3B), suggesting that all injections are able to equally improve surgical outcome. Based on these results and on the elevated PlGF levels in the AC of glaucoma patients, intracameral injection was determined as the most optimal administration route for the following experiments.

### PlGF inhibition improves surgical outcome in a mouse trabeculectomy model

**Figure 4:**
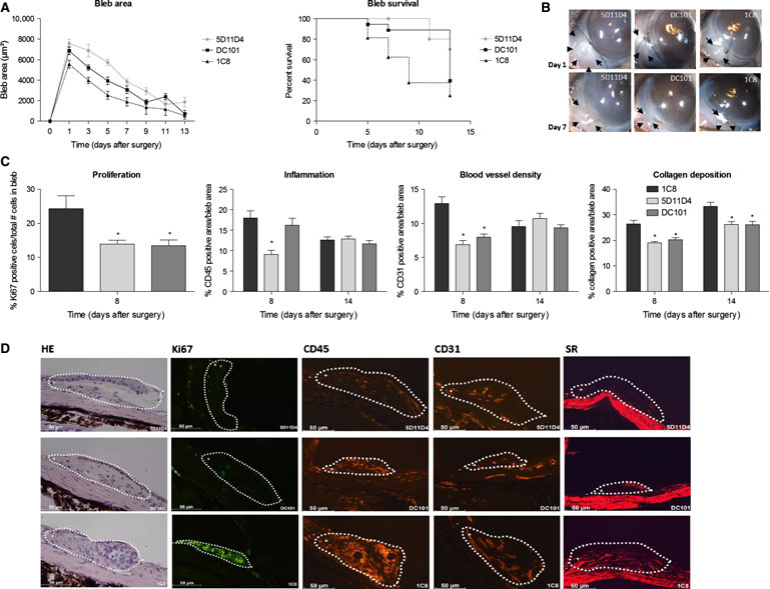
Single intracameral injection of anti-PlGF antibody improves surgical outcome in a mouse trabeculectomy model. (A) Bleb area was significantly larger at each time-point compared with control after administration of the anti-PlGF antibody (*P* < 0.001; *n* = 20), whereas anti-VEGF-R2 antibody did not significantly improve bleb area (*P* = NS; *n* = 20). 5D11D4 also significantly prolonged bleb survival (*P* = 0.002; *n* = 20), while DC101 did not (*P* = NS; *n* = 20). (B) It shows macroscopic post-operative photographs of the blebs on days 1 and 7 after surgery. Arrows: edges of the blebs. (C) Treatment of 5D11D4 significantly decreased the process of inflammation at the filtration site on day 8, whereas DC101 did not. Proliferation, blood vessel density and collagen deposition were reduced after single administration of anti-PlGF and anti-VEGF-R2 antibody (*P < 0.05 compared to 1C8; *n* = 10 per time point). (D) The images show representative pictures of immunostainings of eyes treated with 5D11D4 (upper panels) or with DC101 (middle panels) or control eyes (lower panels), at 8 days after surgery. Edges of the blebs are marked by a dotted line and were indicated as the positive area of analysis (+), whereas the rest of the eye was indicated as the negative area of analysis (−). Scale bar: 50 μm.

To further verify whether PlGF inhibition also affects the process of proliferation and fibrosis *in vivo*, we investigated the therapeutic potential of PlGF inhibition (5D11D4) on wound healing *in vivo* in a mouse trabeculectomy model. A first group of mice received a single antibody injection (5.2 μg) in the anterior chamber (AC) immediately after surgery. To compare the efficacy of 5D11D4 with anti-VEGF therapy, a second group of mice were injected with an anti-VEGF-R2 antibody (DC101; 6.2 μg AC). IOP was measured in all groups, but was found to be similar in the treated and control eyes (1C8; 4.8 μg) at each time-point (*P* = NS, data not shown). This is not unexpected, as these mice do not have IOP elevation at baseline. Successful filtration surgery after antibody administration was evaluated by analysing bleb area and bleb survival at various time-points after surgery. A single injection of the anti-PlGF antibody was able to improve the surgical outcome until day 14 after surgery (Fig. 4B). Indeed, the bleb area was significantly larger at each time-point in the treated eyes compared with the eyes injected with the irrelevant antibody, 1C8 (*n* = 20; *P* < 0.001). Also bleb survival was prolonged after 5D11D4 treatment, as shown in the Kaplan–Meier survival curve, with 25% of the blebs surviving in the control group and 70% of the blebs surviving in the 5D11D4-treated group at 14 days after surgery (*n* = 20; *P* = 0.002). Remarkably, a single injection of the anti-VEGF-R2 antibody failed to significantly improve bleb area (*n* = 20; *P* = 0.08) and bleb survival (*n* = 20; *P* = 0.06) compared with 1C8 treated eyes, although a trend was observed (Fig. 4A).

To evaluate microscopically the effects of anti-PlGF antibody on different phases of wound healing, various (immuno)histological stainings were performed at different time-points after surgery (Fig. 4D). Of note, *in vivo* cell proliferation in the bleb area was only checked at an early time-point (day 8), as it is known that proliferation of different cells (such as endothelial cells and fibroblasts) occurs early in the process of wound healing 4. Analysis of the different processes of wound healing was only performed in the bleb (positive area) and was calculated as a proportion of the total bleb area/total number of cells. Morphometric quantification of a Ki67 staining revealed a significant reduction in the number of all proliferating cells after single 5D11D4 or DC101 administration, being 43% and 45%, respectively, as compared with vehicle injected eyes (*n* = 10; *P* < 0.05). Eyes treated with anti-PlGF antibody showed a significant reduction of 50% in inflammatory area, as compared with 1C8 treated eyes, at 8 days after surgery (*n* = 10; *P* < 0.001). No differences in inflammatory responses were seen after inhibition of VEGF-R2 (*n* = 10; *P* = NS). On post-operative day 8, blood vessel density was reduced after a single 5D11D4 or DC101 administration, with 47% and 38%, respectively, compared with control (*n* = 10; *P* < 0.001). Inflammation and angiogenesis were no longer different on post-operative day 14 after a single administration (*n* = 10; *P* = NS). Collagen deposition was significantly reduced on days 8 and 14 after 5D11D4 administration by 28% and 21% respectively (*n* = 10; *P* = 0.001) and after DC101 injection by 23% and 24% respectively (*n* = 10; *P* = 0.003; Fig. 4C).

**Figure 5:**
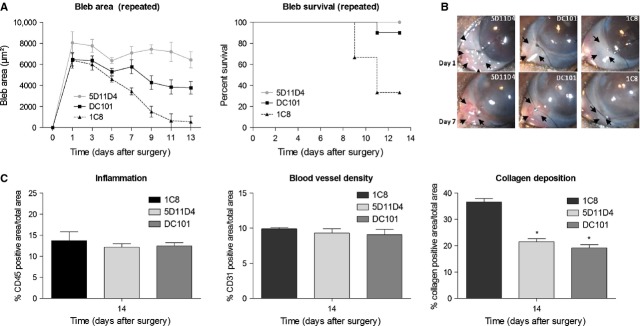
Repeated injections of anti-PlGF antibody improves surgical outcome in a mouse trabeculectomy model. (A) Repeated injections of the anti-PlGF antibody and anti-VEGF-R2 antibody significantly enlarged bleb area (*P* < 0.001 and *P* = 0.005, respectively; *n* = 10) and survival (*P* = 0.006 and *P* = 0.02, respectively; *n* = 10) compared with control. (B) It shows macroscopic post-operative photographs of the blebs on days 1 and 7 after surgery. Arrows: edges of the blebs. (C) Repeated injections of the antibodies did not affect inflammation and angiogenesis. The process of collagen deposition was significantly reduced on post-operative day 14, after repeated 5D11D4 and DC101 administration compared with control (*P < 0.05 compared to 1C8; *n* = 10).

To distinguish whether the observed difference in efficacy of both antibodies was caused by a difference in half-life or by a different working mechanism, repeated intracameral injections of either antibody in the same concentrations were given on day 0, 4 and 10. As expected, repeated administration of 5D11D4 was able to significantly improve the bleb area up until 14 days (*n* = 10; *P* < 0.001) and to prolong bleb survival after filtration surgery compared with 1C8, as shown in the Kaplan–Meier survival curve (*n* = 10; *P* = 0.006). Repeated injections of DC101 were also able to significantly improve surgical outcome. Indeed, the bleb area was significantly enlarged at each time-point in the treated eyes compared with the control eyes (*n* = 10; *P* = 0.005) and bleb survival was significantly prolonged (*n* = 10; *P* = 0.02; Fig. 5A and B). Mice were killed 14 days after surgery and quantification of immunohistochemical stainings showed no effect on inflammation and angiogenesis in the eyes treated with 5D11D4 or DC101 in comparison with control eyes (*n* = 10; *P* = NS). Fibrosis on post-operative day 14, however, was significantly decreased after repeated antibody administration by 41% and 48%, respectively, compared with control (*n* = 10; *P* < 0.001; Fig. 5C).

Thus, administration of an anti-PlGF antibody could improve the outcome in filtration surgery by reducing post-operative inflammation and angiogenesis during the first post-operative days after surgery, and by affecting collagen deposition at later stages. Furthermore, inhibition of PlGF seems to be more effective than anti-VEGF-R2 treatment in improving surgical outcome through its additional effect on inflammation. Indeed, direct comparison between the two antibodies showed that single (*n* = 20; *P* = 0.02) and repeated (*n* = 10; *P* = 0.005) administration of 5D11D4 significantly improved bleb area, whereas DC101 administration only resulted in a small effect on bleb area until post-operative day 14 (data not shown). No significant differences were seen in bleb survival (*P* = NS; data not shown).

## Discussion

In previous studies, we have shown that neither selective nor non-selective VEGF inhibition can reduce the inflammatory response in a rabbit trabeculectomy model 14–15, possibly because of an up-regulation of another VEGF family member, PlGF 20,21. Here, we now revealed that aqueous PlGF levels were indeed significantly increased at different time-points after glaucoma surgery in this rabbit model. This can be explained by the release of PlGF by Tenon fibroblasts and possibly other cells, such as endothelial and inflammatory cells. These cells are also important in the post-operative process of wound healing and are known to express PlGF 24–25. Moreover, bevacizumab treatment was able to enhance this post-operative PlGF up-regulation within the first week after glaucoma surgery. It is known from previous studies that inflammation peaks within the initial days post-surgery in eyes treated with bevacizumab 14. Therefore, we hypothesize that the up-regulated levels of aqueous PlGF after bevacizumab administration might form an explanation why anti-VEGF-therapy was not sufficient to reduce the inflammatory response in our trabeculectomy model 14–15. To further investigate whether PlGF inhibition might influence the post-operative wound healing process through its known anti-angiogenic, anti-inflammatory and possibly anti-fibrotic properties, we used a previously characterized monoclonal antibody (clone 5D11D4) against murine PlGF, produced at ThromboGenics NV. As the available neutralizing antibodies to PlGF did not cross-react with rabbit PlGF (data not shown), a recently developed mouse model for filtration surgery was set up 32–33 to mimic wound healing after glaucoma surgery.

Placental growth factor is a pleiotropic molecule and is known to stimulate endothelial cell growth and migration 24 and the process of fibrosis 39. Although the anti-proliferative effect on endothelial cells by anti-PlGF antibody is well described 26, the effect on Tenon fibroblasts is still unknown. We showed that administration of anti-PlGF (5D11D4) and anti-VEGF-R2 (DC101) to primary cultures of MTF significantly reduced cell number, whereas the irrelevant antibody (1C8) did not induce any differences in cell proliferation. Remarkably, no dose–response effects of the antibodies on PlGF- and VEGF-induced cell proliferation were seen. This can possibly be explained by the secretion and autocrine regulation of PlGF and VEGF by these cells, as described in literature 40,41. We indeed showed that exogenous growth factors only modestly increased the growth of Tenon fibroblasts. These results are in line with those reported by Cianfarani *et al*., who also observed a modest increase in dermal fibroblast proliferation at 48 hrs after PlGF administration, without a dose–response effect 39. We also revealed that MTF are producing VEGF and PlGF, which can bind to the VEGF receptors, known to be expressed on these cells. Moreover, both growth factors are reported to be expressed at relatively high level by fibroblasts 14–43. Therefore, we believe that adding exogenous PlGF and VEGF to fibroblasts may have a relatively limited effect on proliferation because of this endogenous production, which possibly explains the absence of a dose–response effect.

The mouse model of glaucoma surgery was used to confirm the anti-proliferative and anti-fibrotic properties of the anti-PlGF antibody *in vivo*. In this mouse model, the most common reason for bleb failure is scarring and fibrosis 32; therefore, bleb area and survival were investigated as an indication for the fibrotic wound healing response. The intracameral administration route of the PlGF antibody was chosen rather than the subconjunctival and intravitreal route. Although we showed that the three ways of injection of the PlGF inhibitor were able to equally improve surgical outcome, the rationale of this approach is the fact that we found elevated intracameral levels of PlGF in glaucoma patients. This suggests a role of intracameral PlGF in the wound healing process. Anti-PlGF treatment at the time of surgery should therefore aim at preventing PlGF binding to its receptors, but also at preventing PlGF release into AH. An intracameral delivery of the PlGF inhibitor allows blocking this aqueous PlGF. Furthermore, the injected anti-PlGF subsequently passes through the constructed channel, under the flap and into the bleb, where it can also prevent receptor-binding and exert its anti-angiogenic and anti-fibrotic actions. Moreover, subconjunctival injections have the disadvantage of disturbing and stretching the blebs, which may stimulate inflammation and fibroblast activation. Finally, there are also advantages of an intracameral injection over an intravitreal injection. The former can be easily performed with a blunt cannula through the paracentesis already available. Complications associated with intravitreal injections, such as retinal detachment, are thus avoided 44.

We showed that a single intracameral injection of anti-PlGF was effective in improving the surgical outcome, by increasing bleb area and survival until 14 days after surgery, compared with irrelevant antibody 1C8. The pleiotrophic working mechanisms of the anti-PlGF antibody were revealed by immunohistochemistry and analysis showed that overall cell proliferation and angiogenesis was reduced during the initial days of healing and fibrosis at later stages. Importantly, besides affecting proliferation, angiogenesis and fibrosis, the anti-PlGF antibody also had an anti-inflammatory effect in the process of wound healing. PlGF has indeed been described as a chemo-attractant for pro-angiogenic inflammatory cells *via* its binding to VEGF-R1 25. Anti-PlGF attenuates the tissue infiltration by blood-borne macrophages and thereby importantly contributes to angiogenesis by secreting angiogenic factors 45. Moreover, an increase in macrophages also leads to enhanced scar formation in tumour models 26. Remarkably, single administration of 5D11D4 showed to be more potent than the anti-VEGF-R2 antibody in improving bleb area. To elucidate whether the observed difference in efficacy was caused by a difference in half-life or by a different working mechanism of both antibodies, repeated injections on days 0, 4 and 10 were given. Indeed, the systemic half-life of DC101 (4.21 days) was significantly shorter than that of 5D11D4 (7.75 days) 26, which suggests identical properties for the intraocular half-life. Repeated injections revealed that both antibodies were able to improve surgical outcome. Anti-PlGF administration seemed even more effective than inhibition of VEGF-R2. Indeed, a direct comparison of the clinical outcome showed significant differences in bleb area. In contrast to anti-PlGF therapy, anti-VEGF-R2 was able to reduce neovascularization and fibrosis during the wound healing process, but it failed to inhibit infiltration of inflammatory cells. This is consistent with the fact that inflammatory cells do not express VEGF-R2 46, whereas endothelial cells 47 and Tenon fibroblasts do 14. Therefore, we hypothesize that the difference in efficacy of both antibodies could be explained by a different working mechanism, rather than their difference in half-life. Importantly, our results are in keeping with previous studies that showed that anti-PlGF antibody (5D11D4) could reduce angiogenesis and inflammation in preclinical tumour 26 and age-related macular degeneration models 28, whereas inhibition of VEGF-R2 only had an effect on neovascularization. So, administration of anti-PlGF antibody can improve the surgical outcome by reducing the post-operative processes of wound healing, and might be possibly more effective than inhibition of VEGF, because of its additional effect on inflammation.

Although we showed that anti-PlGF treatment was effective in targeting different phases in the process of wound healing, it remains necessary to broaden the therapeutic approach for filtration failure. Treatment with a single anti-angiogenic agent may indeed lead to drug resistance, because of up-regulation of other growth factors. This is based on escape mechanisms *via* induction of an angiogenic rescue programme. It is known that PlGF levels are increased up to 10-fold in various tumour models after VEGF inhibition 20,21. Therefore, a combination of anti-VEGF and anti-PlGF would be a possible option to reduce the escape mechanism and to affect the three most important wound healing phases: inflammation, angiogenesis and collagen deposition. Although it was initially thought that the post-embryonic role of VEGF was restricted to a few processes for which angiogenesis is critical, such as the female reproductive cycle 13, it is becoming obvious that VEGF acts as a pleiotrophic growth factor essential for different physiological processes, such as maintenance of the adult vasculature in many organs 48,49 and neuronal survival 51. As such, because of its essential role in blood vessel formation and maintenance and in neuronal survival, inhibiting VEGF with antibodies at high doses might create severe side effects. PlGF, on the other hand, is an important player in angiogenesis, but only in pathological processes like cancer and inflammation, rather than physiological angiogenesis. Therefore, blocking PlGF with an antibody does not affect the normal vasculature. Furthermore, anti-PlGF was previously reported to enhance the efficacy of VEGF inhibitors 26. Therefore, combination of the optimal dose of anti-PlGF with a suboptimal dose of anti-VEGF (which does not induce any side effects) would probably lead to additional inhibition of scar formation compared with monotherapy of either. Of note, it has been suggested that a lower dose of anti-VEGF might sensitize the vessels for anti-PlGF. Indeed, a study of Van de Veire *et al*. in a mouse model of CNV showed that addition of anti-PlGF allowed a fourfold reduction in the anti-VEGF dose without losing its efficacy 28. Further comparative studies will be necessary to investigate whether this is also true in filtration surgery. The pleiotrophic and complementary mechanisms of anti-PlGF suggest that anti-PlGF may be useful as adjunctive to VEGF inhibition. As such, combining these therapeutic agents may allow reducing their doses, while improving the safety profile of the antiscarring treatment.
